# Genetic diversity of HA1 domain of heammaglutinin gene of influenza A(H1N1)pdm09 in Tunisia

**DOI:** 10.1186/1743-422X-10-150

**Published:** 2013-05-16

**Authors:** Awatef El Moussi, Mohamed Ali Ben Hadj Kacem, Francisco Pozo, Juan Ledesma, Maria Teresa Cuevas, Inmaculada Casas, Amine Slim

**Affiliations:** 1 National Influenza Centre-Tunis, Unit Virology, Microbiology Laboratory, Charles Nicolle’s Hospital, Tunis, Tunisia; 2 National Influenza Centre-Madrid, Influenza and Respiratory Viruses Laboratory, Instituto de Salud Carlos III, Madrid, Spain; 3 Faculty of Medicine of Tunis, Tunis El Manar University, Tunis El Manar, Tunisia

**Keywords:** Antigenic site, Glycosylation site, Heammaglutinin, Influenza A(H1N1)pdm09, Phylogeny

## Abstract

We present major results concerning isolation and determination of the nucleotide sequence of hemagglutinin (HA1) of the pandemic (H1N1)pdm09 influenza viruses found in Tunisia. Amino acid analysis revealed minor amino acid changes in the antigenic or receptor-binding domains. We found mutations that were also present in 1918 pandemic virus, which includes S183P in 4 and S185T mutation in 19 of 27 viruses analyzed from 2011, while none of the 2009 viruses carried these mutations. Also two specific amino acid differences into N-glycosylation sites (N288T and N276H) were detected. The phylogenetic analysis revealed that the majority of the Tunisian isolates clustered with clade A/St. Petersburg/27/2011 viruses characterized by D97N and S185T mutations. However it also reveals a trend of 2010 strains to accumulate amino acid variation and form new phylogenetic clade with three specific amino acid substitutions: V47I, E172K and K308E.

## Introduction

The incidence of recurring epidemics is primarily attributed to the high frequency of mutational changes in the hemagglutinin (HA) and neuraminidase (NA) major surface glycoproteins. The hemagglutinin protein, responsible for viral uptake into host epithelial cells, is also the antigen targeted by antibodies. The hemagglutinin, a trimeric surface glycoprotein that binds the viral receptor and promotes fusion and penetration from low-pH endosomes, is the principal surface antigen on influenza virions [1]. HA presents conserved and variable epitopes, but neutralizing antibodies against the latter dominate the response to immunization and infection [2]. The HA1 subunit of the H1N1 hemagglutinin protein consists of the globular head and contains four major antibody binding sites: Sa, Sb, Ca1, and Ca2, [3,4]. There are determined on the basis of the assessment of antigenic variations of virus mutants [5,6]. Mutational changes (antigenic drift) at these four sites are thought to be driven by selective antibody pressure, which can produce novel strains capable of evading the immunologic response. Because of the high morbidity and mortality due to influenza epidemics, monitoring of the accumulated antigenic variations in circulating influenza viruses is important for predicting epidemics, severity, and for the design of future vaccines [7]. Glycosylation at antigenic sites is an important mechanism of immune evasion by influenza virus [2,8,9]. In fact, the well-known seasonal drift of influenza virus antigenicity accounts for the absence of long-term immune protection in previously infected individuals. In this study we try to present major mutation found in Tunisian pandemic strains.

## Materials and methods

### Sources of specimens

Between May 2009 and January 2011, influenza throat swab specimens were collected from patient in the sentinel centre and patient hospitalized in the hospital in different region in Tunisia. The criteria included patients with a fever >37.8°C accompanied by cough or sore throat. Throat swabs were taken within 72 hours of the onset of symptoms, placed in viral transport media (Vircell, Spain^®^), and delivered to the National Influenza Centre at microbiology laboratory of Charles Nicolle’s Hospital Tunis.

### Sequencing of viral RNA genome

Viral RNA was extracted from 140 μl of clinical sample using the QIAmp Viral RNA Mini kit^®^ (QIAGEN). In order to detect and assign the A(H1N1)pdm09 strains isolated from patients, a real-time PCR assay was used CDC protocol [10]. A representative number of influenza viruses were genotypically characterized by analysis of the nucleotide sequence of partial haemagglutinin HA1 chain (931 nucleotide residues). All viruses analysed were amplified and sequenced according to the protocol of National Influenza Centre Madrid [11]. Mega 4.0 software [12] was used to analyze variations in antigenic sites, virulence-related sites, and glycosylation sites of influenza A (H1N1)pdm09 viruses. Analysis of the phylogenetic relationship was based on nucleotide sequences of HA of the isolated viruses and carried out by the maximum-likelihood method against sequences from global isolates. The evolutionary history was inferred by using the maximum likelihood method based on the Kimura two-parameter model [13]. Sequences generated in this study were deposited in the Genbank database with accession numbers: JN037716– JN037761.

## Results

### Amino acid variations in antigenic sites

Amino acid alignment of the HA of Tunisian isolates in 2009–2010 and 2010–2011 seasons against global isolates was performed, and the results are summarized in Table 1. Minor changes were seen at positions L32F, K36R, N38D, R45K, V47I, S71Y/F, S74N, S84N, D86G/N, I96T, E103K, R113K, F114V, K160R, K171R, E172K, T270A, N276H, N294K/S, P297S, I300N, P304Q, K308E, A315S, and G317R (Table 2). Among these amino acid differences, H138Q, S203T, R205K, D222G, S162I, S183P, and S185T substitutions were located within the antigenic HA1 sites (Ca1, Ca2, Sa, and Sb) (Table 3) in several Tunisian sequences studied. The highest number of variations in predicted antigenic sites were observed in Ca1, where S203T was observed in 46 out of 50 (92%) viruses analyzed and R205K in 10 out of 50 viruses (20%). Analysis of other positions of antigenic sites revealed that Ca1 (H138Q) with 1% substitution, and Sa where S162I (1%) to be highly conserved. Whereas Ca2 had a substitution (D222G/E) in 4 out of 50 (8%).

**Table 1:** Strain information of Tunisian influenza A (H1N1)pdm09 positive samples used in this study

**Tunisian strains of inf A (H1N1)pdm09**	**Date of sampling**	**Accession number (GenBank)**	**Clinical information**
A/Tunisia/59/2009	24/12/2009	JN037736	Severe case
A/Tunisia/13391/2009	26/09/2009	JN037716	Mild case
A/Tunisia/15656/2009	27/10/2009	JN037717	Severe case
A/Tunisia/16501/2009	06/11/2009	JN037718	Severe case
A/Tunisia/17053/2009	12/11/2009	JN037719	Severe case
A/Tunisia/18792/2009	25/11/2009	JN037720	Severe case
A/Tunisia/18861/2009	25/11/2009	JN037721	Fatal case
A/Tunisia/18970/2009	25/11/2009	JN037722	Mild case
A/Tunisia/19073/2009	02/12/2009	JN037723	Severe case
A/Tunisia/19112/2009	03/12/2009	JN037724	Fatal case
A/Tunisia/19436/2009	07/12/2009	JN037725	Mild case
A/Tunisia/19530/2009	08/12/2009	JN037726	Mild case
A/Tunisia/19703/2009	11/12/2009	JN037727	Mild case
A/Tunisia/19922/2009	12/12/2009	JN037728	Severe case
A/Tunisia/19966/2009	11/12/2009	JN037729	Severe case
A/Tunisia/20043/2009	12/12/2009	JN037730	Mild case
A/Tunisia/20083/2009	12/12/2009	JN037731	Mild case
A/Tunisia/20108/2009	13/12/2009	JN037732	Mild case
A/Tunisia/20112/2009	14/12/2009	JN037733	Severe case
A/Tunisia/20558/2009	15/12/2009	JN037734	Severe case
A/Tunisia/21001/2009	23/12/2009	JN037735	Fatal case
A/Tunisia/21516/2009	30/12/2009	HQ174255	Mild case
A/Tunisia/1064/2010	*18/01/2010*	*HM590676*	Fatal case
A/Tunisia/197/2011	04/01/2011	CY080589	Mild case
A/Tunisia/200/2011	04/01/2011	JN037748	Severe case
A/Tunisia/217/2011	06/01/2011	JN037755	Mild case
A/Tunisia/331/2011	07/01/2011	JN037758	Severe case
A/Tunisia/422/2011	07/01/2011	CY080590	Severe case
A/Tunisia/755/2011	20/01/2011	JN037759	Mild case
A/Tunisia/757/2011	20/01/2011	JN037761	Mild case
A/Tunisia/932/2011	26/01/2011	JN037760	Severe case (care unit)
A/Tunisia/1010/2011	28/01/2011	JN037737	Severe case
A/Tunisia/1011/2011	28/01/2011	JN037738	Severe case (care unit)
A/Tunisia/1060/2011	28/01/2011	JN037739	Severe case
A/Tunisia/1198/2011	02/02/2011	JN037740	Severe case
A/Tunisia/1411/2011	07/02/2011	JN037741	Mild case
A/Tunisia/1423/2011	07/02/2011	JN037742	Severe case
A/Tunisia/1909/2011	12/02/2011	JN037747	Severe case
A/Tunisia/1870/2011	14/02/2011	JN037746	Mild case
A/Tunisia/1871/2011	18/02/2011	JN037747	Mild case
A/Tunisia/1701/2011	10/02/2011	JN037743	Severe case (care unit)
A/Tunisia/1713/2011	10/02/2011	JN037744	Mild case
A/Tunisia/2133/2011	19/02/2011	JN037749	Severe case
A/Tunisia/2137/2011	19/02/2011	JN037750	Severe case (care unit)
A/Tunisia/2139/2011	21/02/2011	JN037751	Severe case (care unit)
A/Tunisia/2140/2011	21/02/2011	JN037752	Severe case
A/Tunisia/2141/2011	21/02/2011	JN037753	Severe case
A/Tunisia/2144/2011	22/02/2011	JN037754	Severe case
A/Tunisia/2210/2011	21/02/2011	JN037756	Severe case
A/Tunisia/2222/2011	21/02/2011	JN037757	Severe case

**Table 2:** Amino acid change summary of Tunisia H1N1pdm09 isolates from 2009 and 2011 seasons

**Virus strains of influenza (H1N1)pdm09 **	**Residue position in HA (without signal peptide)**																								
	**32**	**36**	**38**	**45**	**47**	**71**	**74**	**84**	**86**	**96**	**103**	**113**	**114**	**160**	**171**	**270**	**276**	**288**	**294**	**297**	**300**	**304**	**308**	**315**	**317**
A/California/07/2009	L	K	N	R	V	S	S	S	D	I	E	R	F	K	K	T	N	N	N	P	I	P	K	A	G
A/TUNISIA/21001/2009	F	-	-	-	-	-	-	-	-	-	-	-	-	-	-	-	-	-	-	-	-	-	-	-	-
A/TUNISIA/16501/2009	-	R	-	-	-	Y	-	-	-	-	-	-	-	R	-	-	-	-	K	-	-	-	-	-	
A/TUNISIA/20558/2009	-	-	D	-	-	-	-	-	G	-	-	-	-	-	-	-	-	-	-	-	-	-	-	-	
A/TUNISIA/2210/2011	-	-	-	K	-	-	-	-	-	-	-	-	-	-	-	-	-	-	-	-	-	-	-	-	-
A/TUNISIA/331/2011	-	-	-	K	-	-	-	-	-	-	-	-	-	-	-	-	-	-	-	-	-	-	-	-	-
A/TUNISIA/197/2011	-	-	-	-	I	-	-	-	-	-	-	-	-	-	-	-	-	-	-	-	-	-	E	-	-
A/TUNISIA/200/2011	-	-	-	-	I	-	-	-	-	-	-	-	-	-	-	-	-	-	-	-	N	-	E	-	-
A/TUNISIA/1713/2011	-	-	-	-	-	F	-	-	-	-	-	-	-	-	-	-	-	-	-	-	-	-	-	-	-
A/TUNISIA/2144/2011	-	-	-	-	-	-	N	-	-	-	-	-	-	-	-	-	-	T	-	-	-	-	-	-	-
A/TUNISIA/1011/2011	-	-	-	-	-	-	N	-	-	-	-	-	-	-	-	-	-	-	-	-	-	-	-	-	-
A/TUNISIA/2140/2011	-	-	-	-	-	-	-	N	-	-	-	-	-	-	-	-	-	-	-	-	-	-	-	-	-
A/TUNISIA/19966/2011	-	-	-	-	-	-	-	-	G	-	-	-	-	-	-	-	-	-	-	-	-	-	-	-	
A/TUNISIA/20108/2009	-	-	-	-	-	-	-	-	N	-	-	-	-	-	-	-	-	-	-	-	-	-	-	-	
A/TUNISIA/422/2011	-	-	-	-	-	-	-	-	-	T	-	-	-	-	-	-	-	-	-	-	-	-	-	-	-
A/TUNISIA/1060/2011	-	-	-	-	-	-	-	-	-	T	-	-	-	-	-	-	-	-	-	-	-	-	-	-	-
A/TUNISIA/20108/2009	-	-	-	-	-	-	-	-	-	-	K	-	-	-	-	-	-	-	-	-	-	-	-	-	
A/TUNISIA/1701/2011	-	-	-	-	-	-	-	-	-	-	-	K	V	-	-	A	-	-	-	-	-	-	-	-	-
A/TUNISIA/2137/2011	-	-	-	-	-	-	-	-	-	-	-	-	V	-	-	-	-	-	-	-	-	-	-	-	-
A/TUNISIA/2133/2011	-	-	-	-	-	-	-	-	-	-	-	-	V	-	-	-	-	-	-	-	-	-	-	-	-
A/TUNISIA/1010/2011	-	-	-	-	-	-	-	-	-	-	-	-	-	-	R	-	-	-	-	-	-	-	-	-	-
A/TUNISIA/1411/2011	-	-	-	-	-	-	-	-	-	-	-	-	-	-	-	A	-	-	-	-	-	-	-	-	-
A/TUNISIA/15656/2009	-	-	-	-	-	-	-	-	-	-	-	-	-	-	-	-	H	-	-	-	-	-	-	-	
A/TUNISIA/20043/2009	-	-	-	-	-	-	-	-	-	-	-	-	-	-	-	-	-	-	S	-	-	-	-	-	
A/TUNISIA/20112/2009	-	-	-	-	-	-	-	-	-	-	-	-	-	-	-	-	-	-	-	S	-	-	-	-	
A/TUNISIA/1011/2011	-	-	-	-	-	-	-	-	-	-	-	-	-	-	-	-	-	-	-	-	N	-	-	-	-
A/TUNISIA/217/2011	-	-	-	-	-	-	-	-	-	-	-	-	-	-	-	-	-	-	-	-	-	Q	-	-	-
A/TUNISIA/2222/2011	-	-	-	-	-	-	-	-	-	-	-	-	-	-	-	-	-	-	-	-	-	Q	-	-	-
A/TUNISIA/1064/2010	-	-	-	-	-	-	-	-	-	-	-	-	-	-	-	-	-	-	-	-	-	-	-	S	-
A/TUNISIA/17053/2009	-	-	-	-	-	-	-	-	-	-	-	-	-	-	-	-	-	-	-	-	-	-	-	S	-
A/TUNISIA/21516/2009	-	-	-	-	-	-	-	-	-	-	-	-	-	-	-	-	-	-	-	-	-	-	-	-	R

**Table 3:** Dynamic changes of amino acid residues at antigenic and N- glycosylation sites of influenza A(H1N1)pdm09-HA in Tunisia

		**Antigenic sites**								
		**Ca**								
**Virus strains**	**HA1**	**Ca1**			**Ca2**	**Sa**		**Sb**	**N-glycosylation**	
	**321**	**138**	**203**	**205**	**222**	**162**	**183**	**185**	**288**	**276**
A/California/07/2009	I	H	S	R	D	S	S	S	T	N
A/Tunisia/59/2009	-	-	-	-	-	-	-	-	-	-
A/Tunisia/15656/2009	V	-	-	-	-	-	-	-	-	H
A/Tunisia/16501/2009	V	-	T	-	-	-	-	-	-	-
A/Tunisia/17053/2009	V	-	T	-	-	-	-	-	-	-
A/Tunisia/18792/2009	V	-	T	-	-	-	-	-	-	-
A/Tunisia/18861/2009	V	-	T	K	-	-	-	-	-	-
A/Tunisia/18970/2009	V	-	-	-	-	-	-	-	-	-
A/Tunisia/19073/2009	V	-	T	-	-	-	-	-	-	-
A/Tunisia/19112/2009	V	-	T	-	-	-	-	-	-	-
A/Tunisia/19436/2009	V	-	T	-	-	-	-	-	-	-
A/Tunisia/19530/2009	V	-	T	-	-	-	-	-	-	-
A/Tunisia/19703/2009	V	-	T	-	-	-	-	-	-	-
A/Tunisia/19922/2009	V	-	T	-	-	-	-	-	-	-
A/Tunisia/19966/2009	V	-	T	-	-	-	-	-	-	-
A/Tunisia/20043/2009	V	-	T	-	-	-	-	-	-	-
A/Tunisia/20083/2009	V	-	-	-	-	-	-	-	-	-
A/Tunisia/20108/2009	V	-	T	-	-	-	-	-	-	-
A/Tunisia/20112/2009	V	-	T	-	E	-	-	-	-	-
A/Tunisia/20108/2009	V	-	T	-	-	-	-	-	-	-
A/Tunisia/20558/2009	V	-	T	-	-	-	-	-	-	-
A/Tunisia/21001/2009	V	-	T	-	-	-	-	-	-	-
A/Tunisia/21516/2009	V	-	T	-	-	-	-	-	-	-
A/Tunisia/1064/2010	V	-	T	K	G	-	-	-	-	-
A/Tunisia/197/2011	V	-	T	-	-	-	-	-	-	-
A/Tunisia/200/2011	V	-	T	-	-	-	-	-	-	-
A/Tunisia/217/2011	V	-	T	K	-	-	-	-	-	-
A/Tunisia/331/2011	V	-	T	-	-	-	-	T	-	-
A/Tunisia/422/2011	V	-	T	-	-	-	P	-	-	-
A/Tunisia/755/2011	V	-	T	K	-	-	-	T	-	-
A/Tunisia/757/2011	V	-	T	K	-	-	-	T	-	-
A/Tunisia/932/2011	V	-	T	-	-	-	-	T	-	-
A/Tunisia/1010/2011	V	-	T	-	-	-	-	T	-	-
A/Tunisia/1011/2011	V	-	T	-	-	-	P	-	-	-
A/Tunisia/1060/2011	V	-	T	-	-	-	P	-	-	-
A/Tunisia/1198/2011	V	-	T	K	-	-	-	T	-	-
A/Tunisia/1411/2011	V	-	T	-	G	-	-	T	-	-
A/Tunisia/1423/2011	V	Q	T	K	-	-	-	-	-	-
A/Tunisia/1909/2011	V	-	T	-	-	-	-	T	-	-
A/Tunisia/1871/2011	V	-	T	K	-	-	-	T	-	-
A/Tunisia/1870/2011	V	-	T	-	-	-	-	T	-	-
A/Tunisia/1713/2011	V	-	T	K	-	-	-	T	-	-
A/Tunisia/1701/2011	V	-	T	-	G	-	-	T	-	-
A/Tunisia/1713/2011	V	-	T	-	-	-	-	-	-	-
A/Tunisia/2133/2011	V	-	T	-	-	-	-	T	-	-
A/Tunisia/2137/2011	V	-	T	-	-	-	-	T	-	-
A/Tunisia/2139/2011	V	-	T	K	-	-	-	T	-	-
A/Tunisia/2140/2011	V	-	T	-	-	-	-	T	-	-
A/Tunisia/2141/2011	V	-	T	-	-	-	-	T	-	-
A/Tunisia/2144/2011	V	-	T	-	-	I	P	-	N	-
A/Tunisia/2210/2011	V	-	T	-	-	-	-	T	-	-
A/Tunisia/2222/2011	V	-	T	-	-	-	-	T	-	-

Several signature amino acids found in 1918-like viruses were observed in sequence data analyzed. Two additional mutations similar to 1918-like virus emerged in 2011 only: Proline at position 183 in four virus sequences from 2011 season (Table 2); and threonine at position 185 in 19 sequences only from 2011 season.

The most common variation compared to the vaccine strain was P83S, which was actually observed in almost of the Tunisian isolates (96%). Furthermore, a high proportion, 98%, of Tunisian strains possessed the variation I321V. It was observed that the remaining virus that retained isoleukine at this position was associated with severe illness, patient developed pneumonia. Most importantly, it was one strain, A/Tunisia/15656/2009 with altered N-linked glycosylation site, due to variation N276H. All but one of the pandemic viruses had retained all the potential 6 N-glycosylation sites. The one exception (A/Tunisia/15656/2009) had lost a glycosylation site with a change at amino acid residue 276 from asparagine (N) to histidine (H).

It was an alteration to preexisting glycosylation site, and none of the Tunisian isolates gained new glycosylation sites. Moreover, It was one strain, A/Tunisia/2144/2011 showed a switch into N-linked glycosylation site (T288N), associated with severe pneumonia. Interestingly, this case has got three more substitutions in the antigenic sites (S203T, S162I and S183P). Another interesting strain, A/Tunisia/1423/2011 contained three substitutions (H138Q, S203T and R205K). This strain was associated with a severe pneumonia in pregnant woman.

Moreover, three viruses possessed the previously identified D222G/E mutation at the Ca2 antigenic site of the HA. Interestingly, three out of four patients with the D222G mutation (75%), suffered from pneumonia and were hospitalized. One of those infections was fatal (33.33%). However, this mutation was also detected in one virus that caused mild illness (25%).

### Phylogentic analysis of H1N1pdm09 viruses from 2009 and 2011

**Figure 1:**
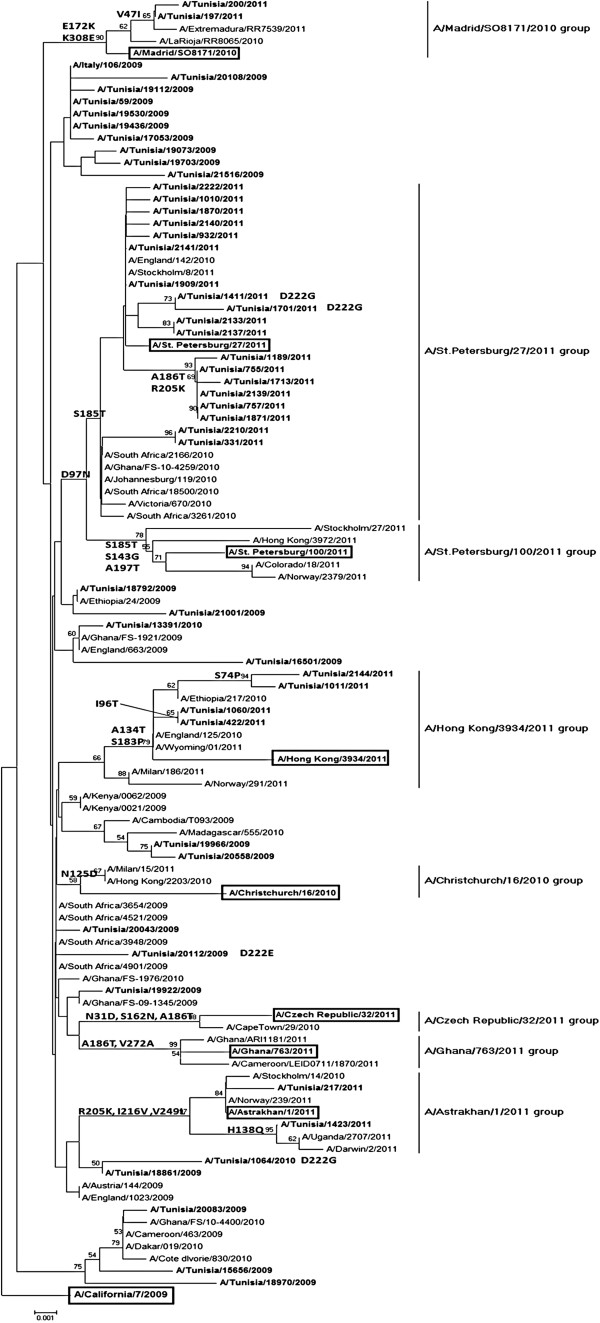
**Phylogenetic relationship of partial length HA sequences of influenza A(H1N1)pdm09 viruses in Tunisia.** The tree was rooted with the vaccine strain A/California/07/2009 (boxed) as outgroup. Branch lengths are drawn to scale. Signature amino acid changes (H1 numbering) are annotated at the nodes of each cluster.

To understand the genetic diversity of influenza A (H1N1)pdm09 in Tunisia, sequencing of 50 HA1 regions of isolates collected in either 2009–2010 season (n = 23) and 2010–2011 season (n = 27) was undertaken (Table 1). Tunisian strains were compared to that of the vaccine strain A/California/7/09 (H1N1) and a number of variations were detected. Substitutions are mentioned in detail in Table 2 and Table 3. Phylogenetic analysis of the HA1 subunit of the HA gene showed that circulating influenza A(H1N1)pdm09 viruses during the season 2010–2011 could be differentiated into eight different and independent genetic groups (Figure 1). This analysis revealed that the Tunisian strains studied were into four clades out of eight defined in the last ECDC report. Moreover, it revealed that the majority (19 of 27) of viruses analyzed clustered with the HA of clade A/St. Petersburg/27/2011 viruses regardless of whether they were from 2011 characterized by D97N and S185T, with additional mutations in minor subclusters such as A186T, K205R and F114V. In A/Astrakhan/1/2011 group we have two strains: A/Tunisia/217/2011 and A/Tunisia/1423/2011 carried D97N, R205K, I216V and V249L substitution. Into this group one strain has an additional mutation H138Q. Third group clustred into A/Hong Kong/3934/2011 clade included four Tunisian strains characterized by A134T and S183P mutations. And two specific mutations (S74N and I96T) only showed in Tunisian viruses. Finally, Most importance of this analysis showed two Tunisian viruses clustred into a new Madrid/SO8171/2010 group defined by E172K, K308E and V47I [14].

## Discussion

Pandemics are believed to arise when a novel avian or swine influenza HA and/or NA are acquired through reassortment between human, swine, and avian influenza viruses or by a non-human virus adapting to efficient human transmission [15]. The current pandemic H1N1pdm09, which is the result of genetic reassortment of multiple gene segments from different lineages, has a known evolutionary history of about a century [16]. Thus, the unpredictability of viral mutations that arise during continued circulation in humans raises questions about antigenic evolution and its impact on future severity of infection, and on vaccine composition. We herein report the characterization of 50 HA genes of the pandemic (H1N1)pdm09 influenza viruses isolated in Tunisia between September 2009 and February 2011, were analysed and their phylogenetic relationships with references strains were showed. We provide evidence that while overall the viruses were fairly homogeneous; there are subtle differences in predicted antigenic epitopes which are potential targets of positive selection pressure.

We further compared the amino acid residues constituting the 7 antigenic sites of A/California/07/2009 with those of the Tunisia isolates. Almost all residues were highly conserved in the Tunisian isolates, however that detailed characterization of the HA gene of H1N1pdm09 has identified some mutations in four antigenic sites (Ca1, Ca2, Sa, and Sb) that are exposed for antibody recognition [16]. The Ca site is proximal to the oligosaccharide at HA1 Asn87, which may interfere with antibody recognition of this region. The sequence data showed that amino acid substitutions were scattered at different loci. However positions 114, 185, 203, 205, 222, and 321 appear the most variable as amino acid residues at these positions vary more than once in the viruses examined. In fact, Melidou et al. 2010 suggest that the occurrence of 321I in severe cases could only be an effect of changing frequency over time rather than an association to severity [17]. An area of largely conserved residues lies between amino acids 280 and 327 and represent part of the stalk structures that supports the globular region of HA1 [18]. Some changes at different positions within the four antigenic domains were observed in sequences from Tunisia. In fact, 11 out 17 (64.7%) Tunisian strains defined by an amino acid change within the haemagglutinin (HA) gene at position 185 (S185T) were from severe cases. Recently, outbreaks varying in severity throughout the UK were caused by a 2009 A(H1N1)pdm09 variant [19]. Analysis of NCBI database sequences revealed the presence of serine at position 183 among H1N1pdm09, while all 1918 (H1N1) viruses have proline at this position. Whether substitution of serine at position 183 has reduced the receptor-binding ability of the (H1N1)pdm09 virus resulting in a less virulent phenotype remains to be determined. It is important to note that 4 of the 2011 viruses from Tunisian severe cases have acquired a proline at this position, which, in combination with additional mutations, could lead to altered pathogenicity [20].

In addition to the variations in the viral HA antigenic sites, observations were made on the number of N-glycosylation sequons in the globular head region of the HA. Since glycosylation can potentially affect the antigenic properties of influenza A virus, we analyzed the changes in the potential N-linked glycosylation sites of the pandemic virus strains. Potential N-glycosylation sites are found in the HA molecule of the A/California/07/2009 virus, six of which reside in the HA1 and the remaining two in the HA2 region. In fact, the absence of glycosylation may influence the function of the glycoprotein, as it is involved in the protein folding, oligomerization, quality control, sorting and transport [17,21]. Among the Tunisian isolates, there were two strains that had variations to glycosylation sites. A/Tunisia/15656/2009 bared the substitution N276H that resulted to the loss of a glycosylation site (NTT). And A/Tunisia/2144/2011 possessed the variation T288N into the glycosylation site. D222G substitution in the HA gene, reported for the first time in influenza A(H1N1)pdm09 virus on November 2009 in Norway [22], has been found again associated to severe patients during the winter 2011 [14]. In the present study, D222G change was detected in 5.2% of severe patients analyzed, percentage similar to that previously reported in 2009–2010 season in Tunisia [23].

Of greater significance is the finding that two of the viruses studied from 2011 shared the amino acid changes V47I, E172K and K308E. In fact, since the genetic characterization algorithms were established in 2010 for pandemic H1N1pdm09 viruses, a new genetic group characterized by these mutations, cited recently, in Spanish study [14]. According to the last ECDC report [24], eight genetic groups were presented whereas the new genetic group reported in this study was indentified as A/Madrid/SO8189/2010. Phylogenetic analysis of these strains showed that, as expected, they belonged to the same clade with the vaccine strain. Further antigenic analysis is needed to assess the characteristics of the rest of the circulating viruses, especially those that possessed variations to antigenic and glycosylation sites of the HA.

However, on the 28th of December, WHO reported that so far all of the circulating pandemic (H1N1) 2009 viruses are antigenically related to A/California/7/2009 [25]. Disease severity is difficult to assess, it can change with different geographic contexts and under different seasonal conditions and may alter as the virus adapts to its new host. Our findings confirm the genetic instability of influenza type A (H1N1) viruses and highlight the importance of continuous molecular surveillance for the effective management of influenza epidemics.

## Abbreviations

ECDC: European surveillance centre for disease prevention and control; WHO: World Health Organization.

## Competing interests

None of the authors has a financial or personal competing interest related to this study.

## Authors’ contributions

AEM proposed the idea, analyzed and interpreted the data and wrote the manuscript, FP revised the manuscript critically for important intellectual content and study design. JL and MTC participated in data analysis and interpretation of data. AS and MHK revised the manuscript and save the final approval of the version to be published. IC and AS obtained funding, administrative, technical, or materiel support and revised the manuscript. All authors read and approved the final manuscript.
